# Hyperbaric Oxygen Therapy Adjuvant Chemotherapy and Radiotherapy through Inhibiting Stemness in Glioblastoma

**DOI:** 10.3390/cimb45100524

**Published:** 2023-10-12

**Authors:** Chun-Man Yuen, Hung-Pei Tsai, Tzu-Ting Tseng, Yu-Lung Tseng, Ann-Shung Lieu, Aij-Lie Kwan, Alice Y. W. Chang

**Affiliations:** 1 Institute of Basic Medical Sciences, National Cheng Kung University, Tainan 701, Taiwan; miconeuron@gmail.com; 2 Division of Neurosurgery, Department of Surgery, Kaohsiung Chang Gung Memorial Hospital, Kaohsiung 833, Taiwan; 3 School of Medicine, College of Medicine, Chang Gung University, Taoyuan 333, Taiwan; 4 Division of Neurosurgery, Department of Surgery, Kaohsiung Medical University Hospital, Kaohsiung Medical University, Kaohsiung 807, Taiwan; carbugino@gmail.com (H.-P.T.); cawaii7992@gmail.com (T.-T.T.); e791125@gmail.com (A.-S.L.); 5 Department of Neurology, Kaohsiung Chang Gung Memorial Hospital, College of Medicine, Chang Gung University, Kaohsiung 333, Taiwan; carbu010200@gmail.com; 6 Department of Surgery, School of Medicine, College of Medicine, Kaohsiung Medical University, Kaohsiung 807, Taiwan; 7 Department of Neurosurgery, University of Virginia, Charlottesville, VA 22904, USA; 8 Department of Physiology, College of Medicine, National Cheng Kung University, Tainan 701, Taiwan; 9 Cheng-Hsing Campus, College of Medicine, National Cheng Kung University, Tainan 701, Taiwan

**Keywords:** hyperbaric oxygen therapy, GBM, radiotherapy, TMZ, stemness

## Abstract

Glioblastoma multiforme (GBM) is the most common and deadliest primary brain tumor in adults. Despite the advances in GBM treatment, outcomes remain poor, with a 2-year survival rate of less than 5%. Hyperbaric oxygen (HBO) therapy is an intermittent, high-concentration, short-term oxygen therapy used to increase cellular oxygen content. In this study, we evaluated the effects of HBO therapy, alone or combined with other treatment modalities, on GBM in vitro and in vivo. In the in vitro analysis, we used a 3-(4,5-Dimethylthiazol-2-yl)-2,5-diphenyltetrazolium bromide (MTT) assay to assess the effects of HBO therapy alone, a colony formation assay to analyze the effects of HBO therapy combined with radiotherapy and with temozolomide (TMZ), and a neurosphere assay to assess GBM stemness. In the in vivo analysis, we used immunohistochemical staining and in vivo bioluminescence imaging to assess GBM stemness and the therapeutic effect of HBO therapy alone or combined with TMZ or radiotherapy, respectively. HBO therapy did not affect GBM cell viability, but it did reduce the analyzed tumors’ ability to form cancer stem cells. In addition, HBO therapy increased GBM sensitivity to TMZ and radiotherapy both in vitro and in vivo. HBO therapy did not enhance tumor growth and exhibited adjuvant effects to chemotherapy and radiotherapy through inhibiting GBM stemness. In conclusion, HBO therapy shows promise as an adjuvant treatment for GBM by reducing cancer stem cell formation and enhancing sensitivity to chemotherapy and radiotherapy.

## 1. Introduction

Glioblastoma multiforme (GBM), a highly aggressive brain cancer that originates in glial cells, is the most common and deadliest form of primary brain tumor in adults. It accounts for approximately 15% of all brain tumors and 50% of all gliomas [1,2], with a global incidence of approximately 3–4 cases per 100,000 individuals [3]. Although the etiology of GBM remains unknown, several risk factors have been identified, including a family history of brain tumors, exposure to radiation, and certain genetic disorders [4,5,6]. Its clinical presentation depends on the location and size of the tumor and can include headaches, seizures, changes in vision or speech, weakness, and cognitive impairment [7,8]. GBM treatment involves a multidisciplinary approach that includes surgery, radiation therapy, and chemotherapy [9,10]. However, due to its aggressive nature, the rate of recurrence is high, with most patients requiring additional treatment [11,12]. Despite the advances in treatment, GBM prognosis remains poor, with a median survival of less than 12–15 months [13].

Hyperbaric oxygen (HBO) therapy is a medical treatment consisting of breathing pure oxygen in a pressurized chamber [14]. The chamber is pressurized to a level higher than that at sea level, and pure oxygen is delivered at a higher concentration than normal atmospheric levels, allowing for improved oxygen delivery to the body’s tissues, which can promote healing and reduce inflammation [15,16]. HBO therapy has been used to treat a variety of conditions, including decompression sickness, carbon monoxide poisoning, non-healing wounds (like diabetic foot ulcers), radiation injuries, and traumatic brain injuries [17,18,19,20,21]. It is also being explored as a potential treatment for stroke, Alzheimer’s disease, and autism [22,23]. Additionally, HBO therapy is sometimes used in cancer treatment to increase the oxygen supply to tumors, which can enhance the effectiveness of radiation therapy and chemotherapy [24,25,26].

This study aimed to investigate the therapeutic effect of HBO therapy, alone or combined with other treatment modalities, by assessing its effects on GBM cell viability and stemness in vitro and in vivo.

## 2. Materials and Methods

### 2.1. Cell Cultures and Treatment Conditions

GBM8401 and T98G GBM cell lines were obtained from the Bioresource Collection and Research Center and American Type Culture Collection cell banks. GBM8401 cells were cultured in Roswell Park Memorial Institute 1640 medium with 10% fetal bovine serum (FBS) in an atmosphere of 5% carbon dioxide (CO_2_) at 37 °C. T98G cells were cultured in Eagle’s minimum essential medium with 10% FBS in an atmosphere of 5% CO_2_ at 37 °C. HBO therapy conditions were induced by incubating cells in an atmosphere of 100% oxygen at 1.5 atm for 1.5 h every day.

### 2.2. Cell Viability Assay

GBM cell viability following HBO therapy alone or combined with temozolomide (TMZ) was assessed using the 3-(4,5-Dimethylthiazol-2-yl)-2,5-diphenyltetrazolium bromide (MTT) assay. GBM8401 and T98G cells (3 × 10^4^ cells per 0.5 mL medium per well) were cultured on a 24-well plate and incubated in an atmosphere of 5% CO_2_ at 37 °C for 24 h. Next, cells were co-cultured with TMZ and/or incubated under HBO therapy conditions for 24, 48, and 72 h and subsequently counted.

### 2.3. Neurosphere Assay

GBM stemness was assessed using a neurosphere assay. In first sphere formation, GBM8401 and T98G cells (100 cells of each) were cultured in a stem cell medium containing 10% Fetal Bovine Serum (FBS), 20 ng/mL of basic fibroblast growth factor, and 20 ng/mL of epidermal growth factor on an ultra-low 24-well plate for 14 days. In secondary formation, we individually dissociated a single cell with 1 mg/mL of collagenase from first sphere formation in GBM8401 and T98G cells, and 100 cells were cultured in a stem cell medium containing 10% Fetal Bovine Serum (FBS), 20 ng/mL of basic fibroblast growth factor, and 20 ng/mL of epidermal growth factor on an ultra-low 24-well plate for 14 days. Five random images were taken, and neurospheres were counted and measured under a microscope at 200× magnification.

### 2.4. Colony Formation Assay

To assess the effects of HBO therapy combined with TMZ, a colony formation assay was used. GBM8401 and T98G cells were seeded on 6-well plates at a density of 100 cells per well for TMZ doses of 0, 5, 10, 15, 20, and 25 μM. After a 10-day incubation, plates were stained with 0.5% crystal violet (Sigma; MFCD00011750) and cell colonies were counted. The number of formed colonies was normalized to the plating efficiency and represented as a cell viability relative to the control.

In addition, a colony formation assay was also used to assess the effects of HBO therapy combined with radiotherapy. GBM8401 and T98G cells were seeded on 6-well plates at densities of 100, 200, 400, 1000, and 10,000 cells per well for radiation doses of 0, 1, 2, 4, and 8 Gy, respectively. A linear accelerator was used to irradiate cells at room temperature. After a 10-day incubation, plates were stained with 0.5% crystal violet (Sigma, St. Louis, MO, USA; MFCD00011750) and cell colonies were counted. The number of formed colonies was normalized to the plating efficiency and represented as a surviving fraction relative to the control. The plating efficiency and surviving fraction were calculated as follows: plating efficiency = (number of colonies formed/number of inoculated cells) × 100%; surviving fraction = number of colonies formed/(number of seeded cells × [plating efficiency/100]).

### 2.5. Animal Model

Sixty immunodeficient and NU/NU nude mice were obtained from the LASCO Laboratory Animal Center (Taipei, Taiwan). All mice were housed at a constant temperature (24 °C) and under regular light/dark cycles between 6:00 am and 6:00 pm, with free access to a standard diet. GBM8401 cells with luciferase (1 × 10^5^ cells in a volume of 5 μL) were injected intracranially into the striata of immunodeficient mice. GBM8401 cells (1 × 10^5^ cells in a volume of 5 μL) were injected intracranially into the striata of NU/NU nude mice. For HBO therapy, mice were placed in an atmosphere of 100% oxygen at 1.5 atm for 1.5 h every day on days 1–21 after tumor cell injection. For chemotherapy, mice were injected intraperitoneally with 10 mg/kg of TMZ every day on days 7–21 after tumor cell injection. For radiotherapy, mice were treated with 2 Gy of radiation three times per week on days 7–21 after tumor cell injection. The protocol of the animal study was approved by the Committee of Institutional Animal Research of Kaohsiung Medical University (IACUC 111223). To assess the effect of HBO therapy in vivo, we evaluated the tumor sizes using in vivo bioluminescence imaging via the Xenogen IVISR Spectrum Noninvasive Quantitative Molecular Imaging System (J&H, Hongkong, China; IVIS Lumina LT 2D) at 7, 14, and 21 days after injection with GBM cells and compared them among the mice treated with HBO therapy, TMZ, radiotherapy, and HBO therapy combined with TMZ or radiotherapy.

### 2.6. Immunohistochemical Staining

At 21 days after tumor cell injection, the brain tissue was removed, and immunohistochemical staining was performed to assess the proportion of CD133-positive cells. Each tissue block was fixed in formalin, embedded in paraffin, and cut into 3 μm thick sections. The sections were deparaffinized, rehydrated, and autoclaved at 121 °C for 10 min in Target Retrieval solution (pH 9.0; Dako; S2368; Glostrup, Danmark) to retrieve the antigens. After 20 min at room temperature, endogenous peroxidase was blocked by adding 3% hydrogen peroxide for 5 min. After being washed with Tris buffer twice, the sections were incubated with anti-CD133 (1:50; Sigma; ZRB1013; St. Louis, MO, USA) antibody for 1 h at room temperature, washed twice with Tris buffer again, and subsequently incubated with horseradish-peroxidase-conjugated secondary antibody for 30 min at room temperature. Finally, the sections were incubated in 3,3-diaminobenzidine (Dako; K5007; Danmark) for 5 min, counterstained with Mayer’s hematoxylin for 90 s, and mounted with Malinol.

### 2.7. Western Blot Assay

For protein extraction, all samples were treated with 200 μL of lysis buffer. Following lysing, 50 μg of protein from each sample was loaded into the wells of a sodium dodecyl sulfate-polyacrylamide gel and subjected to electrophoresis at 50 V for a duration of 4 h. Subsequent to electrophoresis, the proteins were transferred onto poly(vinylidene fluoride) membranes. After a 1 h incubation in a blocking buffer, the membranes were exposed to primary antibodies. These primary antibodies included CD133 (1:200; Sigma; ZRB1013; USA), OCT4 (1:500; Abcam; Cambridge, UK), SOX2 (1:500; Abcam; UK), and β-actin (A5441; 1:20,000; Sigma; USA). Incubation with these primary antibodies was carried out at 4 °C for 16 h. Subsequently, the membranes were incubated with secondary antibodies for 90 min. The secondary antibodies employed were goat anti-rabbit (AP132P, 1:5000; Millipore, Billerica, MA, USA) and goat anti-mouse antibodies (AP124P, 1:5000; Millipore). Specific protein bands were visualized using an enhanced chemiluminescence solution (Western Lightning, 205-14621; Perkin Elmer, Waltham, MA, USA), and image acquisition and analysis were performed using the MiniChemiTM imaging and analysis system (Beijing Sage Creation, Beijing, China).

### 2.8. Statistical Analysis

All statistical analyses were conducted using SPSS 24.0 software (IBM; Armonk, NY, USA). For the quantitative analysis of Western blot data, we utilized the Student’s *t*-test. Kaplan–Meier survival curves were employed to assess survival rates. Additionally, two-way ANOVA was applied for statistical analyses involving time points or dosage effects. A significance level of *p* < 0.05 was considered statistically significant.

## 3. Results

### 3.1. Effect of HBO Therapy on GBM Cell Viability In Vitro

**Figure 1:**
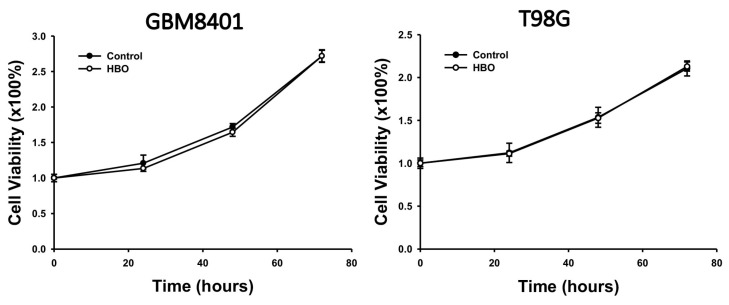
Effect of HBO therapy on GBM cell viability. An MTT assay was used to evaluate the effect of HBO therapy on GBM cell viability. GBM8401 and T98G cells were incubated in an atmosphere of 100% oxygen at 1.5 atm for 1.5 h every day, and cell viability was assessed at 24, 48, and 72 h. GBM, glioblastoma multiforme; HBO therapy, hyperbaric oxygen therapy.

In the MTT assay, we aimed to investigate the impact of HBO therapy on cell viability over a specific time course. Specifically, we assessed cell viability at 24, 48, and 72 h after HBO therapy in GBM8401 and T98G cells compared to their respective pre-HBO therapy values (Figure 1). These experiments were designed to determine whether HBO therapy had any significant influence on the proliferation of GBM cells in vitro.

### 3.2. Effect of HBO Therapy Combined with Chemotherapy or Radiotherapy In Vitro

**Figure 2:**
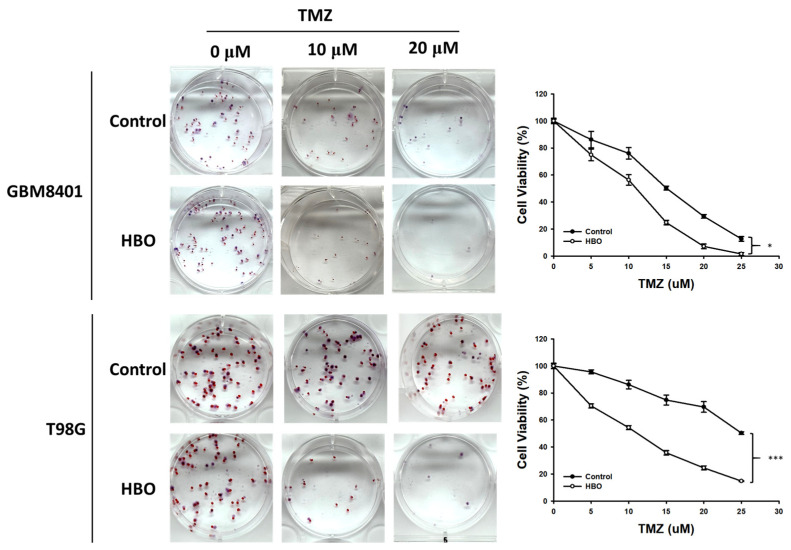
Effect of HBO therapy on TMZ sensitivity of GBM in vitro. To evaluate the therapeutic effect of HBO therapy alone or combined with TMZ, GBM8401 and T98G cells incubated with or without HBO therapy were treated with different doses of TMZ (0, 5, 10, 15, 20, and 25 μM) using 0.5% crystal violet. * *p* < 0.05 and *** *p* < 0.001 compared between control group and HBO group. GBM, glioblastoma multiforme; HBO therapy, hyperbaric oxygen therapy; TMZ, temozolomide.

In the colony formation assay, at 14 days after HBO therapy combined with TMZ, the proportion of viable cells was lower than that in the control group for TMZ doses of 0, 5, 10, 15, 20, and 25 μM in GBM8401 and T98G cells (Figure 2). The result obtained from two-way ANOVA showed that the cell viability of the HBO group was significantly lower than that of control group with TMZ in the GBM8401 and T98G cells. These findings indicate that HBO therapy induced adjuvant effects on chemotherapy with TMZ in GBM cells in vitro.

**Figure 3:**
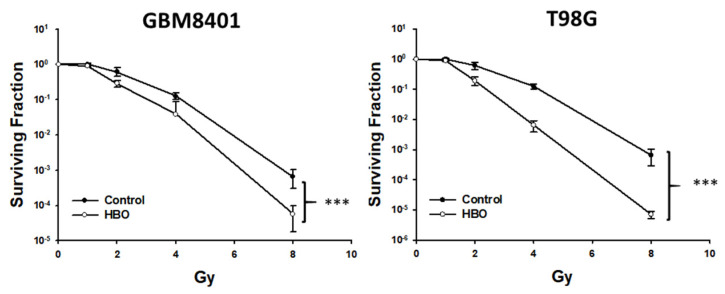
Effect of HBO therapy on radiotherapy sensitivity of GBM in vitro. To evaluate the therapeutic effect of radiotherapy alone or combined with HBO, GBM8401 and T98G cells incubated with or without HBO therapy were treated with different doses of radiation (0, 1, 2, 4, and 8 Gy) following a colony formation assay. *** *p* < 0.001 compared between control group and HBO group. GBM, glioblastoma multiforme; HBO therapy, hyperbaric oxygen therapy.

In the colony formation assay, at 10 days after HBO therapy combined with radiotherapy, the surviving fraction was lower than that in the control group at radiation doses of 4 and 8 Gy in the GBM8401 cells and at radiation doses of 2, 4, and 8 Gy in the T98G cells (Figure 3). Furthermore, the statistical analysis using a two-way ANOVA revealed that these observed differences in the surviving fractions between the treatment groups and control group were statistically significant. This analysis strengthens the conclusion that HBO therapy, when combined with radiotherapy, has a significant adjuvant effect on GBM cells in vitro.

### 3.3. Effect of HBO Therapy on GBM Stemness In Vitro and In Vivo

**Figure 4:**
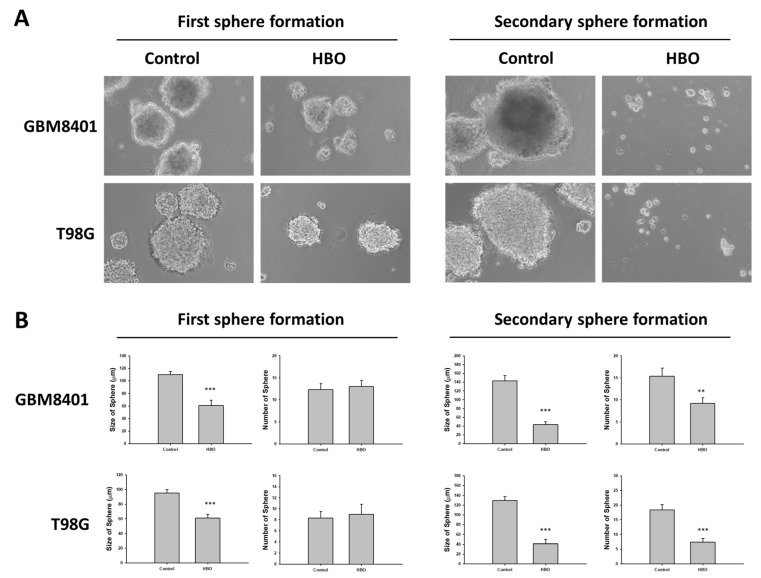
Effect of HBO therapy on GBM stemness in vitro. A neurosphere assay was used to evaluate the effect of HBO therapy on GBM stemness in vitro. The number and size of neurospheres at first and secondary sphere formation were measured in GBM8401 and T98G cells treated or untreated with HBO therapy. (**A**) Microscopic images of neurospheres in GBM8401 and T98G cells treated or untreated with HBO therapy (magnification ×100); (**B**) Box plots of the number and size of neurospheres measured with or without HBO therapy in GBM8401 and T98G cells. ** *p* < 0.01 and *** *p* < 0.001 compared between control group and HBO group. GBM, glioblastoma multiforme; HBO therapy, hyperbaric oxygen therapy.

**Figure 5:**
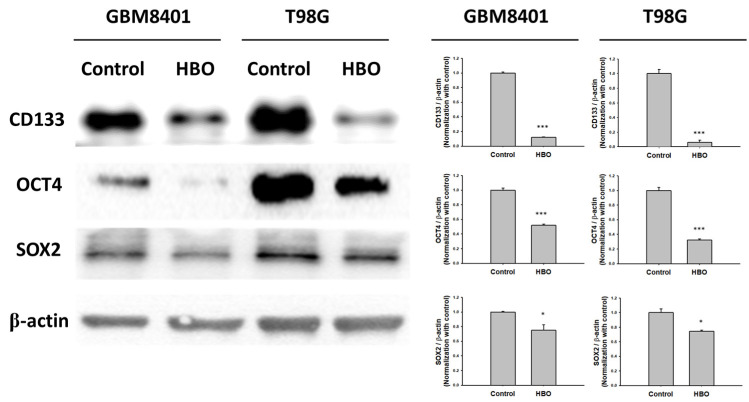
The protein expression of biomarker for GBM stemness in GBM8401 and T98G. The protein expression and relative intensity for CD133, OCT4, and SOX2 determined using Western blot in GBM8401 and T98G cells. * *p* < 0.05 and *** *p* < 0.001 compared between control group and HBO group.

In the neurosphere assay (Figure 4A), the first sphere size after HBO therapy was significantly smaller than that in the control group in the GBM8401 and T98G cells (Figure 4B); however, no significant differences were found in terms of the number of spheres (Figure 4B). These findings indicate that HBO therapy decreased the ability of GBM cells to form cancer stem cells (CSCs) but did not induce cell death. However, the secondary sphere formation assay revealed reductions in both sphere size and number in the HBO group, further supporting the conclusion that HBO therapy impacted the formation of CSCs. The cells had a reduced capacity to form both larger and smaller secondary spheres after exposure to HBO therapy. In addition, we conducted a Western blot analysis to further investigate the impact of HBO therapy on the secondary sphere formation process, specifically focusing on the stemness-related biomarkers CD133, OCT4, and SOX2. The results revealed that in both GBM8401 and T98G cells, the protein expression levels of CD133, OCT4, and SOX2 were significantly lower in the HBO group compared to the control group. This observation indicates that HBO therapy not only influenced the size and number of secondary spheres but also led to reduced protein expression of the critical stemness markers CD133, OCT4, and SOX2 in GBM cells (Figure 5). These findings collectively suggest that HBO therapy may hinder the formation and maintenance of cancer stem cells (CSCs) in GBM, potentially contributing to its therapeutic effects.

**Figure 6:**
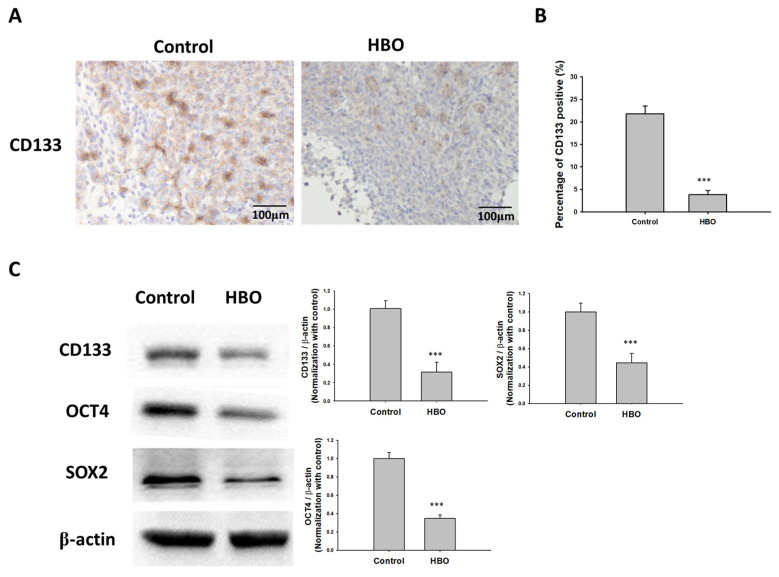
Effect of HBO therapy on GBM stemness in vivo. To evaluate the effect of HBO therapy on GBM stemness in vivo, GBM8401 cells stained with luciferase were injected intracranially into NU/NU nude mice. Mice received HBO therapy every day and were sacrificed at day 21. Immunohistochemical staining was performed to detect CD133-positive cells. (**A**) Microphotographs showing immunohistochemical staining for CD133; (**B**) box plots of the number of CD133-positive cells. (**C**) The protein expression and relative intensity for the stemness biomarkers CD133, OCT4, and SOX2 determined using Western blot assay in GBM from mice. *** *p* < 0.001 compared between control group and HBO group. GBM, glioblastoma multiforme; HBO therapy, hyperbaric oxygen therapy.

In vivo, the results demonstrated significant differences between the control and HBO therapy groups in terms of CD133 expression (Figure 6A). Immunohistochemical staining for CD133 (Figure 6A) showed a notable decrease in CD133-positive cells in the HBO therapy group compared to the control group. This decrease in CD133-positive cells was quantified (Figure 6B), confirming a significantly lower proportion of CD133-positive cells in the HBO group. To gain further insights into the molecular changes associated with these findings, we conducted Western blot analysis (Figure 6C). This analysis encompassed the evaluation of the protein levels of CD133, OCT4, and SOX2. Remarkably, the Western blot results revealed that the HBO therapy group exhibited markedly reduced protein expression levels of CD133, OCT4, and SOX2 when compared to the control group. These combined observations strongly support the notion that HBO therapy has the potential to inhibit the formation and maintenance of cancer stem cells (CSCs) in vivo.

### 3.4. Effect of HBO Therapy Alone and Combined with Chemotherapy or Radiotherapy In Vivo

**Figure 7:**
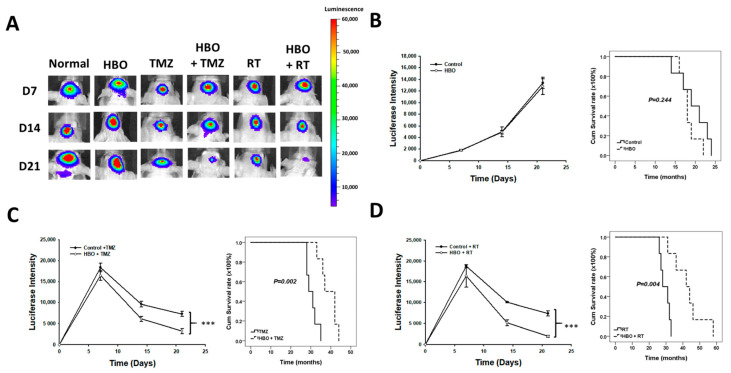
Effect of HBO therapy on TMZ and radiotherapy sensitivity of GBM in vivo. To evaluate the therapeutic effect of HBO therapy alone or combined with TMZ or radiotherapy in vivo, GBM8401 cells stained with luciferase were injected intracranially into NU/NU nude mice. Mice received HBO therapy every day, TMZ every day, and/or radiotherapy three times every week, and they were sacrificed at day 21. At days 7, 14, and 21, in vivo bioluminescence imaging was used to measure luciferase intensity. (**A**) In vivo bioluminescence images obtained at days 7, 14, and 21 with or without conducting HBO therapy, TMZ, and radiotherapy; (**B**) luciferase intensity and survival time comparison between the control and HBO therapy groups; (**C**) luciferase intensity and survival time comparison between the TMZ and HBO therapy + TMZ groups; (**D**) luciferase intensity and survival time comparison between the RT and HBO therapy + RT groups. *** *p* < 0.001 compared between control group and HBO group. GBM, glioblastoma multiforme; HBO therapy, hyperbaric oxygen therapy; TMZ, temozolomide; RT, radiotherapy.

In the in vivo bioluminescence imaging results depict various treatment groups, including the control group, HBO therapy alone, TMZ therapy alone, HBO therapy combined with TMZ, radiotherapy (RT) alone, and HBO therapy combined with RT (Figure 7A). In Figure 7B, the bioluminescence imaging quantification results and a survival rate comparison between the control group and the HBO therapy group are shown. A two-way ANOVA analysis revealed that there were no significant differences in bioluminescence imaging intensity between these two groups (*p* = 0.244). Moreover, the mean survival time for the control group was 19.667 ± 1.542 days, while the HBO therapy group exhibited a mean survival time of 18.333 ± 0.843 days, with no statistically significant difference observed. The results of bioluminescence imaging quantification and survival rate comparison between the TMZ therapy group and the HBO + TMZ therapy group are shown in Figure 7B. Two-way ANOVA analysis demonstrated that the HBO + TMZ group had significantly lower luciferase intensity compared to the TMZ group. Furthermore, the mean survival time for the TMZ group was 30.5 ± 1.118 days, while the HBO + TMZ group exhibited a significantly extended mean survival time of 39 ± 1.751 days (*p* = 0.002). Finally, the results of bioluminescence imaging quantification and survival rate comparison between the RT therapy group and the HBO + RT therapy group are shown in Figure 7D. According to the two-way ANOVA analysis, the HBO + RT group showed significantly lower luciferase intensity compared to the RT group. Additionally, the mean survival time for the RT group was 29.5 ± 1.176 days, whereas the HBO + RT group displayed a substantially longer mean survival time of 42.833 ± 3.781 days (*p* = 0.004). These findings collectively suggest that HBO therapy induces adjuvant effects in both chemotherapy and radiotherapy in the context of GBM in vivo.

## 4. Discussion

In the present study, we explored the impact of hyperbaric oxygen (HBO) therapy on glioblastoma multiforme (GBM) cells both in vitro and in vivo. Our investigations began by assessing GBM cell viability, wherein HBO therapy was found to have no significant effect on cell proliferation. In the combination therapies, we delved into the synergy between HBO therapy and chemotherapy (TMZ)/radiotherapy. Notably, our findings indicated that HBO therapy acted as a potent adjuvant, enhancing the effectiveness of both TMZ and radiotherapy, resulting in reduced cell viability in GBM cells grown in vitro. Additionally, we investigated the influence of HBO therapy on GBM stemness. We discovered that HBO therapy decreased the ability of GBM cells to form cancer stem cells (CSCs) in primary and secondary neurosphere formation assays. Western blot analyses substantiated these observations by revealing reduced protein levels of crucial stemness markers, such as CD133, OCT4, and SOX2, suggesting that HBO therapy hindered CSC formation and maintenance. Finally, in our in vivo experiments, we found that HBO therapy led to a significant decrease in CD133-positive cells, further corroborated by Western blot results showing decreased levels of CD133, OCT4, and SOX2 proteins in the treated groups. This indicates that HBO therapy has the potential to inhibit the formation and maintenance of CSCs in vivo.

CSCs are a small subpopulation of cancer cells that have the ability to self-renew and differentiate into multiple cell types, similar to normal stem cells [27,28]. CSCs are thought to be responsible for tumor initiation, proliferation, and resistance to conventional cancer treatments [29,30]. Therefore, targeting CSCs is an important strategy for developing effective cancer therapies. Our study has shed light on the potential of HBO therapy in this context. By reporting our observation of a reduction in the ability of GBM cells to form cancer stem cells (CSCs) following HBO treatment, we present a novel avenue for intervention. This finding underscores the promise of HBO therapy as an adjunctive approach for GBM, as it appears to hinder the formation and maintenance of CSCs, which are notorious for their role in tumor growth and therapy resistance. Additionally, our research demonstrates that HBO therapy enhances the sensitivity of GBM to conventional cancer treatments such as temozolomide (TMZ) and radiotherapy (RT). This synergistic effect holds substantial clinical implications, as it suggests that HBO therapy, when used in combination with standard treatments, could improve therapeutic outcomes for GBM patients.

Hypoxia-inducible factor-1 alpha (HIF-1α) is a transcription factor that plays a key role in the response of cancer cells to hypoxia [31]. HIF-1α is overexpressed in many types of cancer and has been linked to the survival and self-renewal of CSCs [32,33]. HBO therapy has been shown to regulate the expression of HIF-1α in cancer cells [34] and affect the behavior of CSCs in several ways [35]. By increasing oxygen supply, HBO therapy reduces the hypoxic conditions that promote HIF-1α expression and CSC survival and self-renewal [36], resulting in the downregulation of HIF-1α and a reduction in the survival and self-renewal of CSCs. In our study, HBO therapy decreased the size of neurospheres but did not affect their number. Moreover, HBO therapy did not reduce GBM cell viability. These findings suggest that HBO therapy attenuated GBM stemness but did not induce cell damage.

Additionally, HBO therapy has been shown to enhance the efficacy of some chemotherapy drugs [34,37] as well as the efficacy of radiotherapy among patients with solid tumors [38,39]. The presence of hypoxia in solid tumors reduces their sensitivity to conventional treatment modalities, such as ionizing radiation, and HBO therapy can help overcome this treatment resistance by increasing tumor oxygenation levels [38]. By increasing blood levels of oxygen, both that bound to hemoglobin and that in the plasma, HBO therapy allows for greater oxygen diffusion distances, effectively reoxygenating previously hypoxic cells. This improved oxygenation can enhance the cytotoxic effects of radiotherapy, leading to improved local control of solid tumors. In our study, HBO therapy induced TMZ and radiotherapy sensitivity both in vitro and in vivo.

However, HBO therapy also has disadvantages and limitations that should be considered. First, HBO therapy can have adverse effects, such as hyperoxic seizures and barotrauma, which may limit its clinical application [40]. Second, the administration of HBO therapy can be a complex and potentially dangerous procedure, making it challenging to implement in standard radiotherapy clinics [41,42]. Third, without tumor-specific oxygen delivery, HBO therapy may not effectively target hypoxic tumor tissue, leading to potential side effects due to oxygen toxicity [36,41].

## 5. Conclusions

HBO therapy did not increase the ability of proliferation of GBM cells in vitro, but it did reduce their ability to form CSCs. In addition, HBO therapy increased the sensitivity of GBM cells to chemotherapy and radiotherapy, both in vitro and in vivo. Therefore, HBO therapy may be considered as an adjuvant therapy to chemotherapy and radiotherapy for patients with GBM.

## Data Availability

Not applicable.
